# Uptake, Partitioning, and Accumulation of High and Low Rates of Carbamazepine in Hydroponically Grown Lettuce (*Lactuca sativa* var. *capitata*)

**DOI:** 10.3390/plants14142165

**Published:** 2025-07-14

**Authors:** Emily R. Stamm, Cade Coldren, Clinton Williams, Catherine Simpson

**Affiliations:** 1 Department of Plant and Soil Science, Texas Tech University, Lubbock, TX 79409, USA; ers0086@auburn.edu; 2 Department of Horticulture, Auburn University, Auburn, AL 36849, USA; 3 Department of Natural Resources Management, Texas Tech University, Lubbock, TX 79409, USA; cade.coldren@ttu.edu; 4 Arid Lands Agricultural Research Center, U.S. Department of Agriculture, Maricopa, AZ 85138, USA; clinton.williams@usda.gov

**Keywords:** hydroponics, pharmaceutical, compounds of emerging concern, growth effects, wastewater, drought, water security, deep-water culture, controlled environment agriculture

## Abstract

As potable water becomes limited, alternative water sources, such as reclaimed wastewater, for crop irrigation have gained attention. However, reclaimed wastewater for irrigation may expose edible crops to compounds of emerging concern (CECs), which may include pharmaceutics, hazardous waste, and volatile substances. Of these CECs, carbamazepine (CBZ) is of particular interest because only 7% of CBZ is filtered out during traditional wastewater treatment processing methods. Two trials were designed to evaluate the uptake and partitioning of CBZ in lettuce grown in a deep-water culture system (DWC) at low and high concentrations. The first trial (0 µg L^−1^, 12.5 µg L^−1^, 25 µg L^−1^, and 50 µg L^−1^) of CBZ had few effects on lettuce (*Lactuca sativa* var. *capitata*) growth, and low concentrations of accumulated CBZ were found in lettuce tissues. As a result, increased concentrations of CBZ were used in the second trial (0 mg L^−1^, 21 mg L^−1^, 41 mg L^−1^, and 83 mg L^−1^). Greater amounts of CBZ accumulated in plant tissues and the application of higher rates of CBZ negatively affected the growth and overall health of the lettuce. Further research is needed to determine the impacts of CECs on plant uptake and growth, as well as the environmental conditions.

## 1. Introduction

As water security becomes a threat to agricultural production, a proposed alternative water source is reclaimed wastewater; however, it may contain compounds of emerging concern (CECs) that are persistent in wastewater. This becomes a further concern, as agricultural crops irrigated with alternative water sources take up and accumulate these CECs [1,2,3]. Potential health effects of water contaminated with CECs on humans and animals are unknown; however, one study suggests that normal consumption of edible crops irrigated with CEC-contaminated water is likely to not a health threat [1]. In order to evaluate the potential health risks associated with consuming crops irrigated with CEC-contaminated water, further research is required. In addition to differences in uptake, partitioning, and metabolism by plants, some of these CECs can be persistent and difficult to remove during traditional wastewater treatment practices [1,4,5]. Different CECs also have different rates of environmental persistence, degradation in wastewater treatment systems, and sorption properties [1,6,7]. By studying the uptake and partitioning of these different CECs, researchers will be able to evaluate the risks associated with CEC-contaminated irrigation water.

A CEC of particular interest is carbamazepine (CBZ), which has been used since the 1970s in the U.S. CBZ is a pharmaceutical commonly used for conditions such as ADHD, Bipolar I, and trigeminal neuralgia [8,9,10]. As a result of its tricyclic dibenzoazepine structure with three rings, it is persistent in the environment, and most wastewater treatment facilities are ill-equipped to remove CBZ, as well as other CECs [5,11,12,13,14,15,16]. For example, only 7% of CBZ is filtered out during traditional wastewater treatments [17,18]. Removal rates of persistent compounds range from 1 to 69%, with only one study removing compounds at rates higher than 70% using tertiary treatments [19,20]. Additionally, it may not adsorb to solids in wastewater or soils, requiring advanced oxidation treatments for removal or degradation in wastewater treatment facilities [12,21,22,23]. An experiment sampled 1052 locations in 104 countries and found that the detection frequency of CBZ was 62%, indicating the ubiquity of common pharmaceuticals, such as CBZ [24]. In numerous scientific articles, CECs have been found to affect aquatic life [25,26,27,28,29]. The impacts of tramadol, an analgesic, on the behavior of native European fish indicated that behavioral changes were correlated with the concentration of tramadol in the fish brain [25]. When fish were exposed to environmentally relevant concentrations of methamphetamine, changes in the swimming patterns and velocity were observed [26]. However, the concentrations of CBZ found in wastewater influent and effluent typically range from 207 ng L^−1^ to 5 µg L^−1^. Given the persistence and pervasiveness of CBZ in alternative water sources, it is important to understand how CECs such as CBZ behave in modern agricultural systems, particularly those that rely on hydroponic cultivation under controlled conditions.

Controlled environment agriculture (CEA) production systems use hydroponics for the production of leafy greens and other high-value horticultural crops, with advantages such as improved water use efficiency, year-round cultivation, decreased pest pressures and disease, and the production of high-yielding crops [30,31,32,33]. They are also being evaluated to determine their potential for creating more circular systems that minimize inputs, as well as waste [30,31,32,33]. By using reclaimed wastewater as an irrigation source for hydroponics, water conservation efforts can be realized to a greater degree [30,31,32,33]. However, the presence of persistent CECs in reclaimed wastewater poses a challenge to sustainable water use, and there are limited numbers of studies that explore the uptake and partitioning of wastewater-originating CECs in hydroponically produced crops [5,32,34,35]. This represents a critical gap in the literature, especially when considering the increasing adoption of water-conserving CEA systems such as hydroponics. Most studies on CECs in wastewater focus on soil-based systems, leaving the behavior of CECs in soilless environments underexplored. In one of the few available studies, CBZ, along with twelve other pharmaceuticals, were applied in a hydroponic nutrient solution at a concentration of 50 ng mL^−1^, resulting in variable partitioning of these compounds in different lettuce tissues. Their findings suggest that molecular weight, size, and sorption properties affected partitioning of compounds in plant tissues [36]. The literature on CEC uptake and partitioning suggests that the uptake of CECs follows similar routes to nutrients and other solutes [1,2,16,34,35,36,37,38]. For CBZ, it is assumed that the molecular weight plays an important role in uptake [8,9,10]. The molecular weight of CBZ (263.26 g mol^−1^) is between the molecular weight of sucrose (342.3 g mol^−1^) and glucose (180.16 g mol^−1^), both of which are required for plant functions [9,39]. When compared to a similar pharmaceutical, lamotrigine, the results demonstrated that lamotrigine had higher accumulation in roots, while CBZ had lower uptake in roots but higher transport into shoots [40]. Despite having similar uses, molecular weights, and water solubility, the results indicate that compound affinity to different tissues can affect partitioning; however, the effects of different concentrations have not been explored with regards to translocation [40]. The uptake of compounds into plant tissues could also be due to the chemical properties of the CECs, environmental conditions, physiological properties of the plant, and the chemical properties of the growing media [14,15,36,37,41,42]. Research investigating the effects of biochar amendments in soil produced results that demonstrated that biochar-amended soils can be effective in removing CECs from soil [37,42]. Additionally, the results from research using four types of biochar indicated that while biochar removes the CECs, it does not reduce the concentrations of nutrients in solution [42]. Ion trapping plays a significant role in plant uptake of compounds and is influenced by the pK_a_, logK_ow_, and pH of the compounds [3,43]. As a nonionic compound, CBZ is able to translocate throughout the plant passively; however, ion trapping can influence the uptake of CBZ and other nonionic compounds [3,43]. The rate of uptake of a compound can also be influenced by its dissociation behavior, which depends on the compounds pH [3,43]. Furthermore, the type of production system may significantly influence the uptake and partitioning of CECs due to differences in matrix properties of soil and water. These discrepancies in CEC partitioning across plant tissues and production systems underscores the need for more targeted research, particularly in hydroponic systems, where direct root exposure to nutrient solutions may enhance uptake. For example, one study found that soil organic matter was negatively correlated to CBZ uptake and sandy soils, and hydroponic systems showed higher CBZ accumulation in cucumber tissues [1]. The higher accumulation of CBZ in hydroponic production compared to soil production is due to the direct contact the roots have with the nutrient solution in a hydroponic system [5,34]. From research on CBZ uptake in cucumbers, the authors suggested that CBZ is translocated by water mass flow into source organs, such as older leaves [1]. Because CBZ follows the same pathway as water to source organs, it does not accumulate in the roots [1]. Another study analyzed the absorption of CBZ and diclofenac in a hydroponics system, and the results indicated that CBZ uptake was concentrated in the leaves, where diclofenac was concentrated in the roots [5]. Timing of exposure also plays a role in uptake and accumulation of different compounds. Results from a study of thirteen pharmaceuticals, all applied at the same concentration of 50 ng mL^−1^, showed that within 48 h of application, CBZ accumulated in the shoots more than in the roots, likely due to the plant system prioritizing vegetative growth [40]. This further illustrates the uptake and variability of CECs in plant systems and the need for additional research to understand their dynamics in different production systems.

Despite growing interest in alternative water sources and CEA, few studies have examined how varying concentrations of CBZ are taken up and partitioned in lettuce (*Lactuca sativa* var. *capitata*) over a full growth cycle in hydroponic systems. Lettuce is an ideal model crop for studying the fate and transport of CECs, especially in CEA systems such as aeroponics, deep water culture (DWC), ebb and flow, nutrient film technique (NFT), and aquaponics [30,35,44,45,46,47]. These systems do not require large volumes of water for production in comparison to field systems, are more conducive to increased quality, and are easily adaptable to available water sources. For this experiment, DWC was chosen due to its versatility and ease of nutrient and pharmaceutical application [5,32,34]. While CBZ has been found in different plant tissues, only a few studies have explored uptake and partitioning of CBZ in lettuce grown in different concentrations in DWC systems. Even fewer studies have sampled plants over the entire growing period. Therefore, this study was designed to investigate the uptake and tissue-specific partitioning of CBZ at high and low concentrations in a DWC system, with sampling conducted throughout the entire growing cycle to capture temporal dynamics.

## 2. Materials and Methods

### 2.1. Experimental Setup

This experiment was conducted in the Texas Tech University Greenhouse and Horticultural Gardens (33.58, −101.88) in Lubbock, TX, USA. During the first trial, temperatures were monitored during germination and growing for 13 weeks, from June (25 June 2021) to September (30 September 2021). The greenhouse temperature ranged from 17.56 °C to 36.19 °C, and humidity ranged from 21.5% to 100%. During trial 2, plants were germinated and grown for 9 weeks, from November (8 November 2022) to January (12 January 2023). The temperature ranged from 14.28 °C to 24.39 °C, and humidity ranged from 47.12% to 74.91%.

Both trials 1 and 2 were set up in the same way, with minor differences. The deep-water hydroponics system consisted of 7 L containers, with five containers used for each of the four treatments, for a total of 20 containers for each trial. Buttercrunch butterhead lettuce seeds (*Lactuca sativa* var. *capitata*) were sown in rockwool cubes until the first true leaves were visible, then transferred to plastic hydroponic net pots. Using a randomized block design, six plants were placed in each container for a total of 30 plants per treatment and a total of 120 plants for each trial. To monitor the uptake and partitioning of CBZ at regular intervals, one plant from each container was harvested weekly, for a total of 20 plants per treatment per harvest. The containers were aerated and treated with blue pond dye (Sanco Industries, Fort Wayne, IN, USA) to combat excessive algae growth. In the DWC system, reverse osmosis (RO) water was used throughout the experiments in order to reduce the chemical contaminants and salts that may occur in tap water [13]. Water pH was adjusted following each water change to maintain a pH of 5.6–6.0 [48]. Photosynthetically active radiation (PAR), temperature, and electrical conductivity (EC) were measured weekly (Apera Instruments, Columbus, OH, USA). Treatments for both trials were prepared from stock solutions made with CBZ dissolved in less than 1 mL of methanol due to the low solubility of CBZ in water. These stock solutions were then dispensed into the solution reservoirs each week according to concentration treatments.

In the first trial, 2.85 g of fertilizer (Hoagland’s No. 2, Fisher Scientific, Waltham, MA, USA) was added to each reservoir, and 11.4 g was added to each reservoir in the second trial. In the first trial, CBZ was added weekly at the rates of 0, 12.5 µg L^−1^, 25 µg L^−1^, and 50 µg L^−1^, starting the week after transplant (Table 1). The concentrations of CBZ used were selected to reflect a range of environmentally relevant levels, as well as potential scenarios of elevated exposure. These values are based on concentrations reported in the literature, which spans the lower, intermediate, and higher environmental exposure levels [1,34,49]. Because few impacts of CBZ were found on plant growth in trial 1, concentrations were increased in trial 2 to rates of 0, 21 mg L^−1^, 41 mg L^−1^, and 83 mg L^−1^, added starting the week after transplant, and the water was changed weekly (Table 1). All other factors were similar between trials.

### 2.2. Plant Measurements

Measurements of each plant’s height and width were taken weekly, and at the time of harvest, a growth index was calculated according to Seltsam and Owen [50]. At the time of each weekly harvest, measurements of fresh tissues included root weight (g), inner leaf weight (g), outer leaf weight (g), head height (mm), and head width (mm). Inner and outer leaf weights were determined by taking 50% of the plant materials at the time of harvest; the outermost 50% of leaves were the outer leaves, and the innermost leaves were the inner leaves. The inner leaf weight was combined with the outer leaf weight for the total head weight (g). Plants were then stored at −80 °C before further processing and lyophilization. As soon as plant samples were removed from the −80 °C freezer, the samples were lyophilized until each sample was brittle (Harvest Right, North Salt Lake, UT, USA). Each sample was then weighed, finely ground, and again stored at −80 °C until further analyses could be performed.

### 2.3. Carbamazepine Extraction and Analysis

CBZ was extracted from plant tissues according to the methods described by Wu et al., with some modifications [33]. To begin, 0.20 g of lyophilized, ground plant tissue was placed in a 50 mL polypropylene centrifuge tube with 20 mL of methyl tert-butyl ether (MTBE) for each sample. The test tubes were sonicated for 20 min, then placed in a centrifuge for 20 min at 5000–6000 rpm. The supernatant from each sample was filtered and collected in a new tube. The process of sonification and centrifuging was then repeated with the same sample using 20 mL of acetonitrile. The supernatant was filtered and then added to the tube with the MTBE extracts. Once the samples were extracted, they were evaporated at 40 °C under a gentle flow of nitrogen to a volume of 0.5 mL using a sample concentrator (BT Lab Systems, St. Louis, MO, USA). The remaining residue was redissolved in 1 mL of methanol and mixed with 55–60 mL of deionized water, and the test tubes were rinsed with 10–20 mL of ultra-pure or deionized water. This was then passed through 150 mg Oasis HLB cartridges (Waters, Milford, MA, USA), containing hydrophilic N-vinylpyrrolidone and lipophilic divinylbenzene, at 5 mL min^−1^ under vacuum using a solid-phase extraction (SPE) manifold (VWR, Radnor, PA, USA). The samples were sealed with parafilm and shipped to the Arid Lands Agricultural Research Center in Maricopa, AZ, for CBZ analysis using an ultra-performance liquid chromatograph (UPLC).

### 2.4. Statistical Analysis

Data were analyzed using JMP 16.0.0 (SAS, Cary, NC, USA). Where appropriate, an ANOVA was used to determine significant differences at *p* ≤ 0.05. When significant differences were found, mean separation was determined using Tukey’s HSD tests. An initial correlation analysis was performed using the JMP multivariate function with pairwise comparisons. During correlation analysis, multicollinearity was found. Multicollinearity was removed by computing the variance inflation factor (VIF) and by removing factors with a VIF value greater than 2.5. The remaining factors were then used for the final correlation tables.

## 3. Results

### 3.1. Environmental Conditions

During trial 1, the photosynthetic photon flux density (PPFD; mM m^−2^ s^−1^) had a maximum value of 28.47, a minimum of 2.27, and an average of 15.47 mM m^−2^ s^−1^. Environmental solar radiation data were extrapolated from an outdoor environmental weather station at Texas Tech University Quaker Farm (approximately 2.5 miles away) and calculated to reflect greenhouse conditions (data provided by Dr. T. Montague). Solar radiation averaged 3.42 MJ m^−2^, with a minimum of 2.93 and a maximum of 3.77 MJ m^−2^. Relative humidity fluctuated throughout both trials but was generally under 85%. The mean relative humidity was 73%, with the minimum humidity recorded at 64.14% and the maximum humidity recorded at 80.9%. The average temperature was 24.16 °C. The lowest average temperature was 19.53 °C. Overall, for trial 1, the lowest temperature was 17.84 °C, and the highest temperature was 37.12 °C.

In trial 2, the PPFD ranged from 6.50 to 12.27 mM m^−2^ s^−1^, with an average of 10.33 mM m^−2^ s^−1^. In trial 2, the average solar radiation was 5.08 MJ m^−2^, the minimum was 4.27 MJ m^−2^, and the maximum was 6.33 MJ m^−2^. The average relative humidity was 64.65%, with a minimum of 47.12% and a maximum of 74.91%. The average temperature was 20.27 °C, with the highest temperature recorded at 25.84 °C and the lowest single temperature recorded at 13.2 °C.

### 3.2. Trial 1 Results

#### 3.2.1. Plant Growth and Physiology for Trial 1

**Figure 1:**
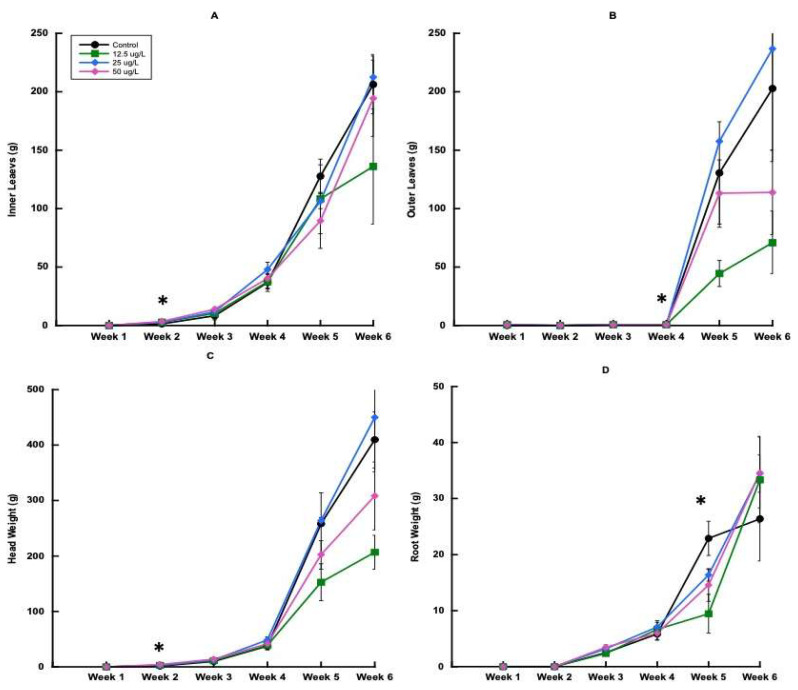
Inner and outer lettuce (*Lactuca sativa* var. *capitata*) leaf weights, head weights, and root weights over time in trial 1. (**A**) Inner leaf weight, (**B**) outer leaf weight for trial 1, (**C**) head weight for trial 1, and (**D**) root weight for trial 1. Error bars represent ±1 standard error of the mean. Significant differences (*p* ≤ 0.05) between treatments at each time point are indicated with (*).

Plant growth significantly increased over time for all parameters in trial 1. For the first and second harvest events, there was a significant effect of applied CBZ concentration on inner leaf weight only (Figure 1A). At week 4, the control had greater outer leaf weight, followed by the 12.5, 25, and 50 mg L^−1^ CBZ treatments. However, after this harvest point, there were fewer differences between the CBZ treatments.

There were also no significant differences in head weight between treatments, with the exception of harvest 2 (Figure 1C). In harvest 2, the 50 mg L^−1^ treatment had the greatest head weight, followed by 25 mg L^−1^, 12.5 mg L^−1^, and 0 mg L^−1^. For root weight in trial 1, there was no significant difference until harvest 5 (Figure 1D). The highest root weight was found in the 0 mg L^−1^ control, followed by 25 mg L^−1^, 50 mg L^−1^, and 12.5 mg L^−1^.

**Figure 2:**
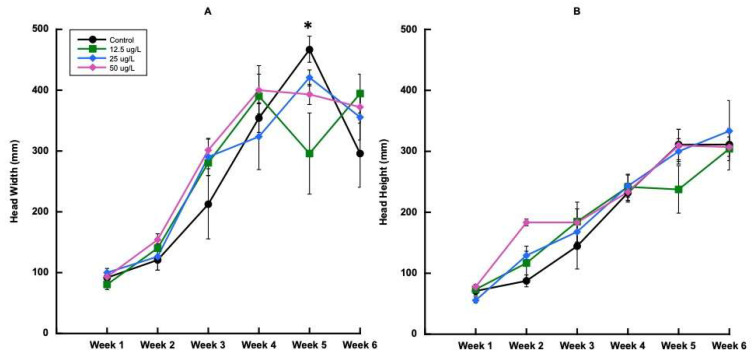
Head width and head height over time during trial 1. (**A**) Head width (mm), (**B**) head height (mm). Error bars represent ±1 standard error of the mean. Significant differences (*p* ≤ 0.05) between treatments at each time point are indicated with (*).

Head width for trial 1 was not significantly different between treatments until the fifth harvest (Figure 2A), where the greatest plant width was found in the 0 mg L^−1^ treatment, then in the 25, 50, and 12.5 mg L^−1^ treatments. No other harvest was significantly impacted by treatment in the first trial. Head height did not show any significant differences between treatments for any of the harvests.

**Figure 3:**
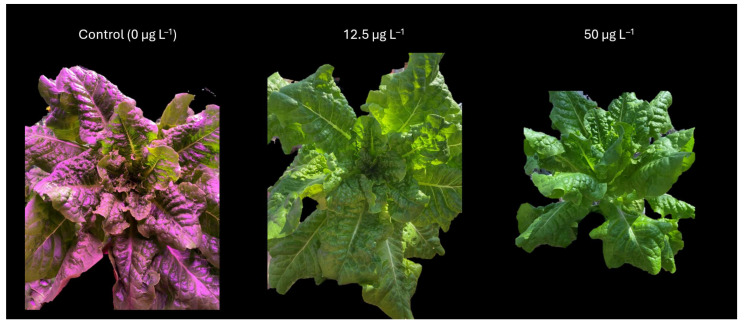
Photos of lettuce (*Lactuca sativa* var. *capitata*) grown in different treatments in trial 1. Photos of plants in the 25 ug L^−1^ treatment were unavailable. Changes in color are due to supplemental lighting and could not be altered. Photos are not to scale.

Plant biomass increased incrementally over time for trial 1. There were no statistical differences between treatments for root weight, inner leaf weight, outer leaf weight, total head weight, and total weight at the final harvest (Figure 3, Table 2).

#### 3.2.2. Carbamazepine Concentrations in Plant Tissues

The concentration of CBZ in the control samples from the final harvest of trial 1 had the lowest total concentration for all plant parts combined. While no CBZ was added to the control treatments, minor amounts of contamination must have occurred due to human error or compound transfer during analysis. The greatest total concentration of CBZ in the samples was observed in the 50 μg L^−1^ treatment, followed by the 12.5 μg L^−1^ treatment and the 25 μg L^−1^ treatment (Table 3). With regard to plant parts, only the treatment concentration significantly affected CBZ found in plant tissues. The 50 μg L^−1^ treatment showed the highest concentrations, but other treatments did not differ significantly. There were no significant differences in CBZ concentrations between the different plant parts, but numerically, the highest applied treatment had the highest concentrations in plant tissues. Furthermore, there was no interaction effect between treatment concentration and plant part.

#### 3.2.3. Correlations Between Carbamazepine and Environmental Factors

To further describe the relationships among CBZ application, environmental conditions, and plant growth, correlations were examined. As expected, most of the growth parameters were positively correlated with each other. Consequently, multicollinearity was found for many factors. The multicollinearity and related factors were removed using VIF, and the remaining factors were then correlated again (Table 4). After removing collinearity for trial 1, the correlation between outer leaf weight, plant width, dry outer leaves, and solar radiation were highly correlated (Table 4). While most of the values were positively correlated with each other, solar radiation was significantly negatively correlated with outer leaf weight and dry outer leaf weight. As expected, as solar radiation increased, PPFD also increased.

### 3.3. Trial 2

In trial 2, larger concentrations of CBZ were applied to determine impacts on growth, uptake, and accumulation over time. To demonstrate the magnitude of concentration differences, the scale of concentrations was changed from µg L^−1^ to mg L^−1^.

#### 3.3.1. Plant Growth and Physiology for Trial 2

**Figure 4:**
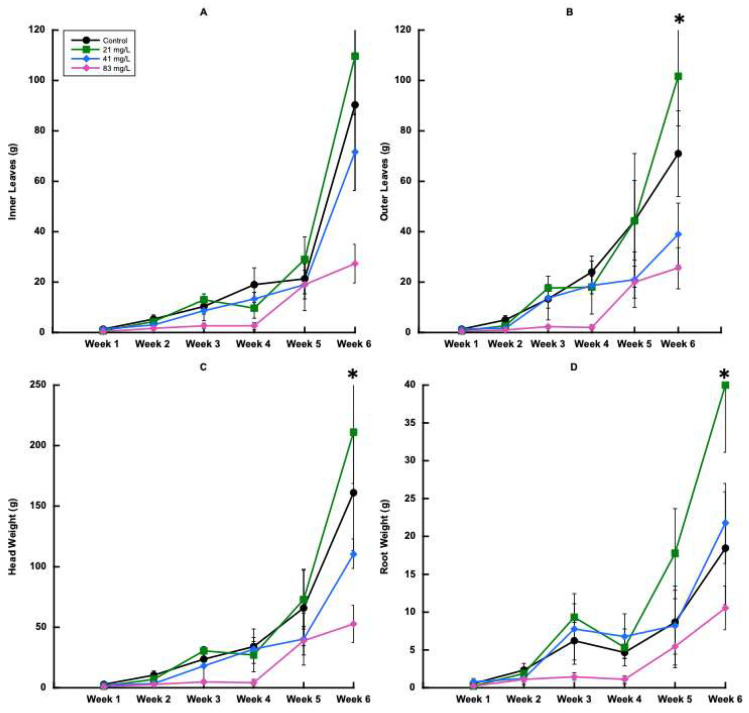
Inner and outer lettuce (*Lactuca sativa* var. *capitata*) leaf weights, head weights, and root weights over time in trial 2. (**A**) Inner leaf weight, (**B**) outer leaf weight, (**C**) head weight, and (**D**) root weight. Error bars represent ±1 standard error of the mean. Significant differences at ≤0.05 are indicated with (*).

**Figure 5:**
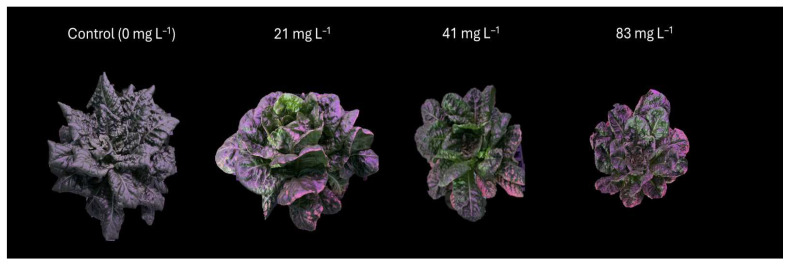
Photos of lettuce (*Lactuca sativa* var. *capitata*) grown in different treatments in trial 2. Changes in color are due to supplemental lighting and could not be altered. Photos are not to scale.

For trial 2, no significant effects of CBZ were apparent on inner leaf weight for any of the harvests (Figure 4A and Figure 5). Furthermore, there were no significant effects of treatment on weight of outer leaves until the last harvest (Figure 4B and Figure 5). By the final harvest, the 21 mg L^−1^ treatment had greater outer leaf weight, followed by the control, 41 mg L^−1^, and 83 mg L^−1^ CBZ treatments.

**Figure 6:**
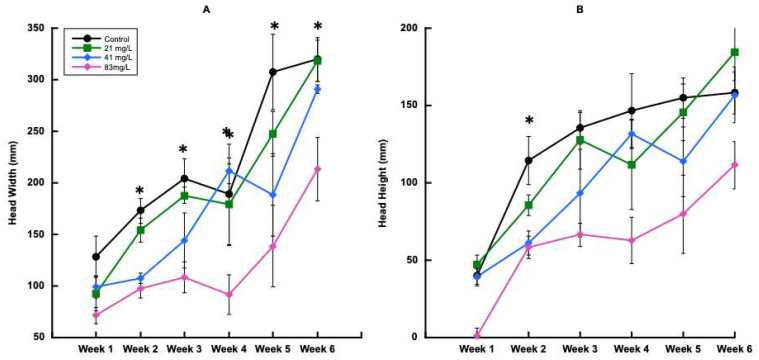
Head width and head height over time during trial 2. (**A**) Head width (mm), (**B**) head height (mm). Error bars represent ±1 standard error of the mean. Significant differences at ≤0.05 are indicated with (*).

There were also no significant differences between treatments until the final harvest for head weight for trial 2, (Figure 6A). At the final harvest, the head weight for the 41 mg L^−1^ treatment was greatest, followed by 0 mg L^−1^, 83mg L^−1^, and finally 21 mg L^−1^. The final harvest was the only harvest to show significant differences in root weight among treatments (Figure 6B). The greatest root weight for trial 2 was found in the 21 mg L^−1^ treatment, then in the 41 mg L^−1^, 0 mg L^−1^, and 83 mg L^−1^ treatments. While the root weight averaged higher in the 21 mg L^−1^ treatment, there was a marked increase in root growth for the 41 mg L^−1^-treated plants at week 4, which continued to increase until week 6. This was likely a result of rapid vegetative growth, as seen in Figure 6A,B.

For harvests 2, 3, 4, 5, and 6, head width was significantly affected by CBZ treatments (Figure 6A). For each of these harvests, head width decreased as the CBZ concentration increased. However, for harvest 2, significant differences were observed, with the 0 mg L^−1^ treatment showing the greatest head height (Figure 6B). At harvest 3, head height was influenced by treatment, similar to harvest 2, where height decreased as treatment concentration increased (Figure 6B). Conversely, harvest 6 plants had greater head height at 21 mg L^−1^, followed by the 0 mg L^−1^, 41 mg L^−1^, and 83 mg L^−1^ treatments (Figure 6B).

Trial 2 showed significant effects of treatment on root weight, outer leaf weight, total head weight, and total weight for the final harvest, as seen in Table 5. The largest plants were found in the 21 mg L^−1^ treatment, followed by the control treatments. The total weight (combined root weight and leaf weights) for the final harvest followed the same order as the total head weight; from greatest to lowest total weight, 21 mg L^−1^, 0 mg L^−1^, 41 mg L^−1^, and 83 mg L^−1^. In trial 2, inner leaf weight was the only factor not significantly affected by treatment. The lowest biomass values were seen in the two highest concentration treatments, indicating the toxic effects of CBZ at these levels.

#### 3.3.2. Carbamazepine

CBZ concentrations (mg L^−1^) within plant tissues varied throughout the six harvests for trial 2 (Table 6). The greatest amount of CBZ found was within the tissues of the highest treatment, 83 mg L^−1^, followed by the 41 mg L^−1^ treatment, the 21 mg L^−1^ treatment, and the control (mg L^−1^). Over the 6 weeks, CBZ uptake varied (Table 6).

For weeks 1–3, the outer leaves were found to have greater amounts of CBZ compared to the inner leaves and roots (Table 6). This was the same for the final harvest, in week 6 (Table 6). During trial 2, harvest 4 had the greatest concentration of CBZ within the plant tissues of the 83 mg L^−1^ treatment, followed by the 41 mg L^−1^ treatment, 21 mg L^−1^, and the control (0 mg L^−1^ treatment; Table 6). The outer leaves had the highest concentration of CBZ when compared to the inner leaves and roots. The inner leaves had the next highest concentration of CBZ, and the roots had the least. This pattern was similar for harvest 5 and harvest 6.

Overall, the average total concentration of CBZ within the plant tissues over all harvests followed the pattern of the 83 mg L^−1^ treatment, which had the greatest concentration of CBZ, followed by the 41 mg L^−1^ and the 21 mg L^−1^ treatments, and lastly the control (0 mg L^−1^) (Table 7). Within the plant tissues, over all 6 harvests, the outer leaves had the greatest concentration of CBZ, followed by the inner leaves, and then the roots, which had the least concentration of CBZ.

#### 3.3.3. Correlations

Similar to trial 1, many of the growth parameters from trial 2 were highly collinear. After collinearity was removed, the remaining variables were height, dry roots, dry inner leaves, solar radiation, air temperature, relative humidity, and total concentration of CBZ in plant tissues (Table 8). Height was significantly positively correlated to dry inner leaf weight but negatively correlated to solar radiation. Solar radiation and air temperature increased proportionally as well. Solar radiation was also the only factor that had a significant relationships with the total concentrations of CBZ in plant tissues, which indicated inverse relationships that were likely indirectly related. Temperature and relative humidity were also positively correlated.

## 4. Discussion

### 4.1. Environmental Influence of Carbamazepine Uptake and Plant Growth

Environmental factors are highly related to plant growth and development [6,31,39,51,52]. Typically, plant growth rates increase with light and temperature to a certain point, after which, increases result in detrimental effects to plant health [53,54]. Other factors that affect plant growth are total solar radiation, quality of light, and relative humidity, particularly if heat or drought stress occur [39,51,55,56]. With increased solar radiation, temperatures can increase depending on season. Optimal temperature ranges vary by crop, but temperatures outside of the optimal temperature range can induce heat stress and affect yield [53,57]. Lettuce is especially sensitive to temperature changes and will adapt by producing leaves with greater leaf area, longer stem lengths, and reduced leaf thickness [52,54,57,58]. A larger leaf area is positively associated with increased photosynthesis and plant uptake [6,53,57]. An additional effect of increased temperature is increased rate of transpiration [6,39,51,52]. Results from a study on the effects of transpiration on uptake demonstrated that transpiration has significant effects on plant uptake of CECs [6].

During the first trial, temperatures were warmer than in the second trial. While the first trial had larger plants overall, fewer effects of CBZ were seen. Enzyme activity is necessary to carry out important physiological processes within plants and tends to speed up at higher temperatures. The optimal temperatures for lettuce production are 25 °C during the day and 19 °C at night. As a result, increased uptake of solutes is often observed under these conditions [39,51,52]. Moreover, increased temperatures enhance the solubility of chemical compounds, which can further increase uptake. The higher temperatures during trial 1 could have affected the plants’ response to CBZ application in lettuce, but this was more likely due to the lower rates of CBZ applied. For each day of trial 1 and trial 2, the temperature was recorded, and the average for each day was calculated. Over the duration of trial 1, the total daily average was 24 °C, while trial 2 had a total daily average of 21 °C, indicating that in trial 1 temperatures were close to optimal levels for lettuce biomass production [48,52]. Yet, another article found that temperatures between 15 °C and 20 °C were optimal for growing lettuce, which may indicate that the plants in trial 2 were under less temperature stressed [53]. However, temperature is not the only factor that influences plant growth. High humidity can reduce transpiration rates and slow the rate of growth [39,51,52]. In these experiments, the seasons did affect relative humidity levels. According to Shibata et al., higher air velocity combined with low relative humidity will cause water stress and stomatal closing [59]. Conversely, Carvalho et al. observed that relative humidity above 85% also caused stomatal malfunctioning, which resulted in water stress [60]. In this study, our results indicate that solar radiation had greater impacts on plant growth, as demonstrated by the correlation analysis, whereas temperature did not.

### 4.2. Plant Growth Effects

Studies have found varying effects of compounds of concern on plant growth and yields, but these effects are highly dependent upon crop species. Shenker et al. showed no differences in biomass when cucumbers were spiked with up to 1000 μg L^−1^ CBZ [1]. However, when exposed to rates over 10,000 mg L^−1^, phytotoxicity was observed [1]. Alternatively, Knight et al. and Carter et al. found that increasing rates of CBZ up to 20 mg kg^−1^ caused visible toxic effects on plants, including leaf edge necrosis in the older leaves [14,16]. Root growth was also reduced in all of the CBZ treatments [16]. This further indicates that the effects of CBZ need to be studied in low and high concentrations, due to its variable impacts on yield and indications that CEC accumulation occurred [5,14,16]. Few negative effects of CBZ rates on lettuce were seen in the first trial, which involved lower rates of CBZ, but detrimental effects of higher rates were found in the second trial. In the first trial, the 25 mg L^−1^ CBZ treatment seemed to have slight stimulating effects on lettuce growth, although these were not statistically significant. In trial 2, it was noted that the control and 21 mg L^−1^ treatments resulted in significantly larger plants, which could indicate that concentrations up to 21 mgL^−1^ were not detrimental to plant growth. The 83 mg L^−1^ CBZ treatment was detrimental to lettuce, and plants were noted as small and appeared unhealthy, exhibiting some phytotoxicity symptoms. Edge necrosis was also observed during both trials, similar to the results from research on CECs in zucchini [14,16]. Tip burn and edge necrosis are also a common physiological problems in greenhouse-produced lettuce, as a result of air circulation and calcium deficiencies [53,59,61]. While there is a possibility that edge necrosis could have been due to environmental and nutritional effects, it is more likely that there was a phytotoxic effect due to the treatment-specific responses [53,61]. Few studies have focused on lettuce as the crop of interest when examining lower or higher concentrations of CBZ in plant tissues, whereas this study looked at higher and lower concentrations of CBZ and how they impacted growth [5,7]. For example, Leitão et al. found that lettuce treated with CBZ at rates of 0.1 mg L^−1^, 1 mg L^−1^, and 5 mg L^−1^ showed an increase in hydrogen peroxide generation, which is commonly used as an indicator of oxidative stress [7]. The resulting plant stress has been demonstrated to affect individual plants differently and has been found to vary between crops and compounds of concern [1,7,34]

Plant uptake and accumulation of different compounds of concern have been found to vary, not only in tissue concentrations, but also in their impacts on plant growth parameters [4]. It is assumed that CBZ is taken up in plant tissues due to its neutral properties, as well as its molecular weight. Additionally, factors that may affect uptake include CBZ’s pKa, pH, ionic strength, biodegradation, and sorption properties [3,5,8,9,10]. González García et al. found that movement of CBZ by evapotranspiration is due to these characteristics, allowing for more accumulation in leaf tissues [5]. Furthermore, Malchi et al. found that nonionic compounds are taken up more readily in roots than ionic compounds, indicating that CBZ should also been taken up easily [3]. Partitioning of CBZ in different plant tissues has been explored to only a limited extent. In an experiment by Shenker et al., the roots of cucumber plants had the least amount of CBZ [1]. Shenker et al. also noted that higher concentrations of CBZ were found in the older leaves, which is similar to our findings, indicating that CBZ concentrations were higher in the outer leaves compared to the inner leaves and roots [1]. Therefore, CBZ likely accumulates within the leaves following the movement of water, transpiration, and factors that increase water uptake and transpiration, such as higher temperatures [3,5].

### 4.3. Carbamazepine Accumulation and Partitioning in Plant Tissues

The use of alternative water sources such as wastewater is common in regions such as Arizona, USA, and Israel, due to limited freshwater availability. Even in treated water, compounds such as CBZ are persistent and resist degradation [1,2,3,13,14,28,29,41]. The presence and concentrations of CECs in food products have been studied to a limited extent, but must be addressed due to the inevitability of their occurrence, especially as water becomes more limited, and alternative sources are sought. Removal of these compounds requires expensive technologies that may not be available, particularly in rural or economically challenged areas. While some processes, such as RO and UV light photodegradation are integrated into newer systems, older infrastructures may not have the capacity to perform advanced removal [13,21,62].

Cross-contamination during sample processing occurred in the first trial, resulting in a secondary extraction from the final harvest tissues. The concentrations of CBZ found in the re-extracted samples followed similar patterns to plant growth responses in the first trial, and the percentage taken up by the plants was much lower than in the second trial. While the highest treatment of 50 µg L^−1^ had the highest concentration of CBZ found within the plant tissues, the remaining treatments, 12.5 µg L^−1^ and 25 µg L^−1^, had significantly lower concentrations. The results from trial 2 show that a larger percentage of CBZ was taken up by the 21 mg L^−1^ treatment compared to the other treatments. The plants in the 21 mg L^−1^ treatment took up 28% of the applied CBZ; the plants in the 41 mg L^−1^ treatment took up 18% of the applied CBZ; the plants in the 83 mg L^−1^ treatment took up 20% of the applied CBZ. These findings may indicate that, at lower concentrations, a greater proportion of CBZ could have been taken up. The concentrations of CBZ in solution may have also affected degradation or metabolism of these compounds, but this was beyond the scope of this study.

While trial 1 showed no significant differences in amount of CBZ accumulation in different tissues, trial 2 did see significant differences. Two separate studies, one using cucumbers and the second using carrots and sweet potatoes, found that the lowest concentration of CBZ was found in the roots of these plants [1,3]. Similar findings by Knight et al. show that CBZ concentrations in the roots of zucchini were lower than the concentrations of CBZ in leaf tissues by a factor of at least 10 [14]. The experiments conducted by Shenker et al., Malchi et al., and Knight et al. support the findings from trial 2 throughout all harvests; the concentration of CBZ accumulated in the root tissues was less than the accumulation of CBZ in the leaf tissues [1,3,14]. This was also the case for all harvests in trial 1, excluding harvest 6. The highest concentration of CBZ within plant tissues during trial 2 was found in the outer leaves of the 83 mg L^−1^ treatment. The trend for CBZ concentrations within plant tissues increased with the amount of CBZ applied. The concentration of CBZ accumulation within plant tissues was the highest for all treatments in the outer leaves, followed by the inner leaves, and lastly the roots. The results of this study support findings from previous literature suggesting that CBZ is translocated with mass flow, accumulating in the source organs rather than the roots.

Furthermore, the factors that influenced CBZ uptake and partitioning varied with the applied concentration. At generally lower concentrations, such as those applied in trial 1, we saw that plant tissues were negatively affected with increasing applied concentrations. However, solar radiation also negatively affected plant tissues. Alternatively, total tissue concentrations in plants that were treated with higher concentrations of CBZ (0, 21 mg L^−1^, 41 mg L^−1^, and 83 mg L^−1^) were negatively impacted by solar radiation. These results indicate that there are indirect effects of solar radiation on plant tissue concentrations of CBZ. Solar radiation reduced plant height, outer leaf weight, and was negatively correlated with total concentrations of CBZ. It is unlikely that solar radiation directly caused decreased concentrations of CBZ, but more likely this is due to indirect impacts of solar radiation that caused or exacerbated phytotoxic effects. This could indicate that higher concentrations of CBZ increased plant sensitivity to solar radiation, or that the negative effects of CBZ were compounded by higher solar radiation. Other researchers have also found that the environment and the applied concentration of compounds affects the uptake and distribution within tissues [1]. During an experiment on the uptake of CBZ by cucumber plants, CBZ was applied at concentrations of 0, 1, 10, 100, 1000, 10,000, and 100,000 µg L^−1^. The authors reported that 1 µg L^−1^ was typically found in effluents [1]. The results from the study showed that CBZ was taken up and had accumulated in edible cucumber plants at rates commonly found in wastewater [1]. Thus, their research further supports evidence that CEC uptake and partitioning in plants is highly variable in different systems, under different environmental conditions, and will vary according to exposure concentrations. Exposure concentrations will also impact yields and the quality of plants being produced, which presents further challenges when irrigating with alternative water sources.

## 5. Conclusions

Overall, variable effects of CBZ on lettuce growth and accumulation were found, which were highly correlated with the rates applied and environmental factors that affect transpiration. Higher rates of applied CBZ negatively affected lettuce growth in concentrations greater than 21 mg L^−1^. Previous research suggests that CBZ can hinder plant growth at higher concentrations, which is supported by the findings in this study. While most environmental scenarios would not consist of such high concentrations as those shown in the second study, the findings in this research highlight that CBZ can bioaccumulate in lettuce over time, potentially reaching notable levels in plant tissues, even with mild or no obvious phytotoxic effects. Additional research is needed to evaluate the broader implications of CEC accumulation in plants, particularly with respect to their effects on plant growth, development, and yield, as well as potential impacts on human health.

## Figures and Tables

**Table 1:** Dose of carbamazepine (CBZ) and the amount added to solution per treatment weekly and the total applied.

	Concentration of CBZ	Dose of CBZ Added (mg)		Amount of CBZ in Solution (mg)	
		Weekly	Total	Weekly	Total
Trial 1 (µg L^−1^)	0	-	-	-	-
	12.5	0.0125	0.075	0.09	5.4
	25	0.025	0.15	0.18	10.8
	50	0.05	0.3	0.36	21.6
Trial 2 (mg L^−1^)	0	-	-	-	-
	21	21	126	151.2	907.2
	43	43	258	309.6	1857.6
	82	82	492	590.4	3542.4

**Table 2:** Average plant fresh biomass (g) at the final harvest date for trial 1.

Trial 1					
Dose Concentration of CBZ (μg L^−1^)	Root Weight (g)	Inner Leaves (g)	Outer Leaves (g)	Total Head Weight (g)	Total Weight (g)
0	26.38	206.10	202.96	409.06	435.44
12.5	32.52	135.98	71.10	207.08	239.60
25	34.44	212.82	236.60	449.42	483.86
50	34.60	194.34	113.80	308.14	342.74
*p*-value	0.80	0.38	0.195	0.072	0.325

**Table 3:** Carbamazepine concentrations (µg L^−1^) after six weeks of growth in trial 1.

Final Harvest *				
Dose of CBZ (μg L^−1^)	Total Concentration (µg L^−1^)	Inner Leaves Concentration (µg L^−1^)	Outer Leaves Concentration (µg L^−1^)	Root Concentration (µg L^−1^)
0	0.27 b	0.00	0.14	0.17
12.5	10.17 b	3.31	4.47	2.38
25	8.62 b	6.18	1.93	2.13
50	73.23 a	24.68	22.80	25.75
*p* concentration	*<0.0001*	*0.0001*		
*p* plant part	N/A	0.725		
*p* concentration × part	N/A	0.819		

* Due to contamination during extraction, only the final harvest was analyzed. Different lowercase letters indicate significant differences between means at *p* < 0.05 for concentration only, italicized text indicates significance at <0.01. No significant effects were seen for plant part or interaction between concentration and plant parts.

**Table 4:** Correlation matrix for trial 1 after removing highly correlated variables. Bold indicates significance at <0.05, italicized text indicates significance at <0.01, and text formatted in both bold and italics indicates significance at <0.001.

	OL	W	DOL	SR	PPFD
**OL**	1.000				
**W**	***0.404***	1.000			
**DOL**	***0.605***	***0.427***	1.000		
**SR**	*−0.340*	*0.327*	***−0.352***	1.000	
**PPFD**	−0.030	0.080	0.090	*0.241*	1.000

Outer leaf weight (g) (OL), width (mm) (W), dry outer leaves (g) (DOL), solar radiation (kW h m^−2^) (SR), photosynthetic photon flux (mmol m^−2^ s^−1^) (PPFD).

**Table 5:** Average plant fresh biomass (g) at the final harvest date for trial 2. Significant differences between means at *p* ≤ 0.05 are shown by different lowercase letters, italicized text indicates significance at <0.01. Numbers without letter designations indicate a lack of significance.

Trial 2					
Concentration (mg L^−1^)	Root Weight (g)	Inner Leaves (g)	Outer Leaves (g)	Total Head Weight (g)	Total Weight (g)
0	18.41 ab	90.40	71 ab	161.4 ab	179.81 ab
21	39.97 a	109.80	101.6 a	211.4 a	251.37 a
41	21.73 ab	71.60	39 b	110.6 ab	132.33 b
83	10.57 b	27.36	25.5 b	52.86 b	63.43 b
*p*-value	*0.036*	0.097	*0.010*	*0.025*	*0.008*

**Table 6:** Total concentration of carbamazepine (mg L^−1^) for trial 2, in different plant tissues, at different times of plant development. Uppercase letters indicate significant differences between means for the interaction of concentration and plant part. Lowercase letters indicate significant differences between concentration treatments; ‘na’ indicates where analysis is not applicable.

Harvest	Treatment Concentration (mg L^−1^)	Total Concentration (mg L^−1^)	Inner Leaves Concentration (mg L^−1^)	Outer Leaves Concentration (mg L^−1^)	Root Concentration (mg L^−1^)
1–3 *	0	8.59 c	5.28 D	2.36 D	3.60 D
	21	29.43 bc	13.98 CD	21.53 CD	6.61 D
	41	46.15 b	19.62 CD	47.09 AB	5.34 CD
	83	82.32 a	33.71 BC	60.66 a	19.62 B
	*p* concentration	*0.0001*		*0.0001*	
	*p* part	*na*		*0.0001*	
	*p* conc. × part	*na*		*0.002*	
4	0	9.16 d	1.30 B	5.70 B	2.16 B
	21	43.76 c	11.04 B	27.68 B	15.95 B
	41	110.67 b	25.62 B	69.71 A	19.17 B
	83	187.79 a	77.32 A	95.10 A	15.37 B
	*p* concentration	*0.0001*	*0.0001*		
	*p* part	*na*			
	*p* conc. × part	*na*	*0.0003*		
5	0	1.76 c	0.30 C	0.93 C	0.54 C
	21	66.28 b	34.15 ABC	24.61 BC	7.51 C
	41	107.03 ab	23.21 BC	60.79 AB	23.02 BC
	83	111.47 a	35.45 ABC	78.70 A	24.20 BC
	*p* concentration	*0.0014*	*0.0001*		
	*p* part	na	*0.0003*		
	*p* conc. × part	na	*0.0117*		
6	0	2.56 c	0.62	0.67	1.28
	21	44.08 bc	9.40	29.74	4.93
	41	87.66 b	28.89	41.81	21.20
	83	153.06 a	54.35	63.36	35.35
	*p* concentration	*0.0004*	*0.0001*		
	*p* part	na	*0.0114*		
	*p* conc. × part	na	0.59		

* Harvests 1–3 were combined due to small sample size. Uppercase letters indicate significant differences between means for the interaction between concentration and plant part. Lowercase letters indicate significant differences between concentration treatments; ‘na’ indicates where analysis is not applicable, italicized text indicates significance at <0.01.

**Table 7:** Average total concentration of carbamazepine (mg L^−1^) for the entire study for trial 2. Uppercase letters indicate significant differences between means for the interaction between concentration and plant part. Lowercase letters indicate significant differences between concentration treatments; ‘na’ indicates where analysis is not applicable.

Dose Concentration of CBZ (mg L^−1^)	Total Average Concentration (mg L^−1^)	Inner Leaves Average Concentration (mg L^−1^)	Outer Leaves Average Concentration (mg L^−1^)	Root Average Concentration (mg L^−1^)
0	6.54 d	3.01 F	2.41 EF	2.23 EF
21	40.40 c	16.09 DE	25.02 CD	8.04 EF
41	73.97 b	22.76 D	53.30 AB	14.81 DEF
83	116.54 a	44.71 BC	71.70 A	22.83 CD
*p* concentration	*0.0001*	*0.0001*		
*p* part	*na*	*0.0001*		
*p* conc. × part	*na*	*0.0001*		

Uppercase letters indicate significant differences between means for the interaction between concentration and plant part. Lowercase letters indicate significant differences between concentration treatments; ‘na’ indicates where analysis is not applicable, italicized text indicates significance at <0.01.

**Table 8:** Correlation matrix for trial 2 after collinearity was removed. Bold indicates significance at <0.05, italicized text indicates significance at <0.01, and text formatted in both bold and italics indicates significance at <0.001.

	HE	DR	DIL	SW	T	RH	TC
**HE**	1.000						
**DR**	0.072	1.000					
**DIL**	***0.689***	0.145	1.000				
**SW**	*−0.325*	0.150	−0.100	1.000			
**T**	−0.172	0.192	−0.197	***0.225***	1.000		
**RH**	0.106	0.126	−0.061	−0.154	***0.580***	1.000	
**TC**	0.823	0.029	0.052	**−0.244**	0.004	0.090	1.000

Height (mm) (HE), dry roots (g) (DW), dry inner leaves (g) (DIL), solar radiation (kW h m^−2^) (SR), air temperature (°C) (T), relative humidity (%) (RH), total concentration (mg L^−1^) (TC).

## Data Availability

The data presented in this study are available on request from the corresponding author. The data are not publicly available due to extent of data and platform limitations.
